# Relationship between exposure to PM2.5 and lung cancer incidence and mortality: A meta-analysis

**DOI:** 10.18632/oncotarget.17313

**Published:** 2017-04-21

**Authors:** Feifei Huang, Bing Pan, Jun Wu, Engeng Chen, Liying Chen

**Affiliations:** ^1^ Department of Family Medicine, Sir Run Run Shaw Hospital, School of Medicine, Zhejiang University, Hangzhou, 310016, P.R. China; ^2^ Department of Colorectal Surgery, Sir Run Run Shaw Hospital, School of Medicine, Zhejiang University, Hangzhou, 310016, P.R. China

**Keywords:** PM2.5, lung cancer, mortality, incidence, meta-analysis

## Abstract

We conducted a meta-analysis to examinine the relationship between exposure to PM2.5 and lung cancer incidence and mortality. In total, 17 studies met our inclusion criteria and provided information necessary to estimate the change in lung cancer risk per 10 μg/m^3^ increase in exposure to PM2.5. The random-effects model was used to estimate the relative risk (RR) for specific PM2.5 values. The meta-estimate for lung cancer risk associated with PM2.5 was 1.11 for mortality (95% CI: 1.05, 1.18) and 1.08 (95% CI: 1.03, 1.12) for incidence. Analyses by continent showed that the meta-estimate for lung cancer mortality associated with PM2.5 was greatest in North America [1.15 (95% CI: 1.07, 1.24)], followed by Asia [1.12 (95% CI: 0.94, 1.35)], and then Europe [1.05 (95% CI: 1.01, 1.10)]. Lung cancer incidence associated with PM2.5 was greatest in Asia [1.09 (95% CI: 1.03, 1.15)], followed by North America [1.06 (95% CI: 1.01, 1.11)], and then Europe [1.03 (95% CI: 0.61, 1.75)]. In subgroup analyses of country, the mortality meta-estimate for developed countries was 1.14 (95% CI: 1.06, 1.23), and for developing countries was 1.03 (95% CI: 1.00, 1.07). The incidence meta-estimate for developed countries was 1.07 (95% CI: 0.96, 1.20), and was similar to that of developing countries, 1.07 (95% CI: 1.06, 1.09). In subgroup analyses of males and females, the meta-estimate for lung cancer mortality associated with PM2.5 was greater for males [1.26 (95% CI: 1.15, 1.40)] than for females [1.17 (95% CI: 0.98, 1.39)]. The meta-estimate for lung cancer incidence associated with PM2.5 was greater for males [1.23 (95% CI: 0.83, 1.81)] than for females [1.15 (95% CI: 1.12, 1.18)]. In subgroup analyses of smoking status, the meta-estimate for lung cancer mortality associated with PM2.5 for former smokers was 1.46 (95% CI: 0.84, 2.55), for current smokers was 1.33 (95% CI: 1.20, 1.49), and for never smokers was 1.16 (95% CI: 1.02, 1.33), respectively. The meta-estimate for lung cancer incidence associated with PM2.5 for former smokers was 1.19 (95% CI: 0.95, 1.50), for never smokers was 1.10 (95% CI: 0.76, 1.59), and for current smokers was 1.03 (95% CI: 0.87, 1.21). The relative risks of a relationship between PM2.5 and lung cancer incidence and mortality were 1.08 (95% CI: 1.03, 1.12) and 1.11 (95% CI: 1.05, 1.18), respectively. These findings will provide some evidence for policy makers and public health practitioners worldwide.

## INTRODUCTION

Lung cancer (LC) is one of the most prevalent and deadliest human cancers. It is estimated that there were 1.825 million LC cases globally in 2012, accounting for 13.0% of all cancer cases, and 1.59 million deaths from LC, accounting for 19.4% of all cancer deaths [1]. Therefore, it is essential to emphasize the importance of LC prevention and knowledge of modifiable risk factors. In October 2013, the International Agency for Research on Cancer (IARC) classified particulate matter (PM) from outdoor air pollution as carcinogenic to humans and causes LC [2]. PM with a diameter less than 2.5 μm (PM2.5) is often used as an indicator of the level of anthropogenic air pollution: the average annual concentrations of PM2.5 currently range from less than 10 μg/m^3^ to over 100 μg/m^3^ worldwide [3]. Evidence suggests that PM2.5 from vehicle emissions significantly increases the risk of LC [4]. In addition, PM2.5 and PM10 were found to be risk factors for LC in mortality and incidence studies [5]. However, we consider incidence and mortality to be different endpoints, and the present meta-analysis was conducted to determine the relationship between PM2.5 and LC incidence and mortality, respectively. To fully characterize such an association, we carried out subgroup analyses defined by continent, country, gender, and smoking status. We also examined the influence of single studies in the overall meta-analysis.

## RESULTS

### Literature selection

**Figure 1:**
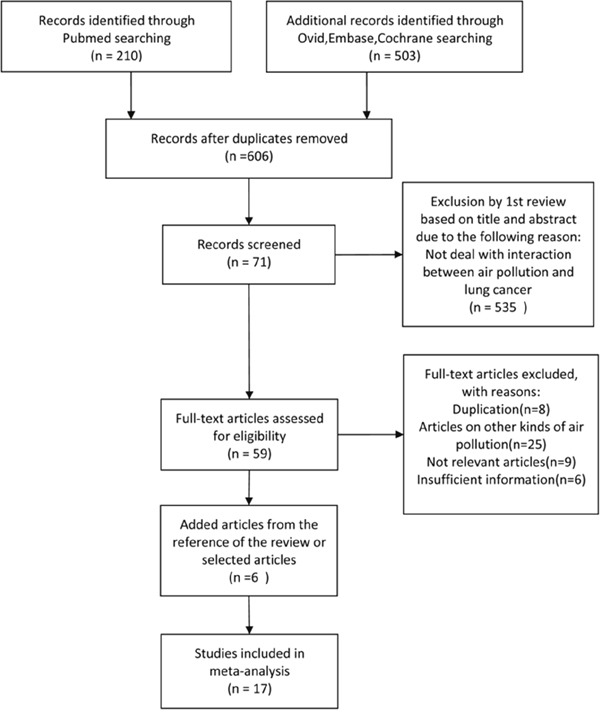
Study selection flow chart

Restricted to human studies, a total of 713 articles were identified from the PubMed (210), Ovid, Embase and Cochrane Library (503) databases. In total, 606 articles remained after the exclusion of duplicate articles. After reviewing the study titles and abstracts, 535 articles which did not investigate the interaction between air pollution and LC were excluded. A full review of the remaining 71 articles identified 11 articles that fulfilled the inclusion criteria. In addition, six articles were identified from the references of selected articles. A total of 17 articles were finally selected (Figure 1).

### Study characteristics

Table 1 summarizes the 17 studies [7–23] (McDonnell et al. 2000; Pope et al. 2011; Hart et al. 2011; Lipsett et al. 2011; Lepeule et al. 2012; Hystad et al. 2013; Puett et al. 2014; Hart et al. 2015; Weichenthal et al. 2016; Beelen et al. 2008; Carey et al. 2013; Cesaroni et al. 2013; Raaschou-Neilsen et al. 2013; Cao et al. 2011; Katanoda et al. 2011; Yorifuji et al. 2015; Guo et al. 2016) included in this analysis. In total, 9 and 8 studies provided estimates of PM2.5 associated LC mortality and incidence, respectively.

**Table 1:** Summary of the characteristics of the studies included in the meta-analysis

Continent	Study ID	Reference	No. of events	Total population	Study period	RR/HR(95%CI)	Study type	Study
North America								
California, USA	1	McDonnell et al.2000	13(mortality)	3,769	1977–1992	1.39(0.79,2.46)	Cohort	AHSMOG
United States	2	Pope et al.2011	3,194(mortality)	794,784	1988–1994	1.14(1.04,1.23)	Cohort	ACS-air pol extend
United States	3	Hart et al. 2011	800(mortality)	53,814	1985–2000	1.18(0.95,1.48)	Cohort	TrIPS
California, USA	4	Lipsett et al. 2011	234(mortality)	73,489	1997–2005	0.95(0.70,1.28)	Cohort	CTS
United States	5	Lepeule et al. 2012	632(mortality)	8,096	1975–2009	1.37(1.07,1.75)	Cohort	Harvard Six Cities Study
Canada	6	Hystad et al. 2013	2,390(incidence)	5,897	1994–1997	1.29(0.95,1.76)	Case-control	National Enhanced Cancer
								Surveillance System Case-Control study
United States	7	Puett et al. 2014	1,648(incidence)	97,865	1998–2010	1.06(0.9,1.24)	Cohort	NHS
United States	8	Hart et al.2015	3,355(incidence)	12,085	1986-2003	1.37(0.86,2.17)	Cohort	NLCS
Canada	9	Weichenthal et al.2016	3,200(incidence)	193,300	1991-2009	1.05(1.00,1.10)	Cohort	Can-CHEC
Europe								
Netherlands	10	Beelen et al. 2008	1,940(incidence)	120,852	1986–1997	0.81(0.63,1.04)	Cohort	Netherlands Cohort study Of Diet and Cancer.
United Kingdom	11	Carey et al. 2013	5,273(mortality)	830,842	2003–2007	1.11(0.86,1.43)	Cohort	Clinical Practice Research Datalink
Italy	12	Cesaroni et al. 2013	12,208(mortality)	1,265,058	2001–2010	1.05(1.01,1.10)	Cohort	Rome Longitudinal Study
European Union	13	Raaschou-Neilsenet al. 2013	2,095(incidence)	312,944	1990	1.39(0.91,2.13)	Cohort	
Asia								
China	14	Cao et al. 2011	624(mortality)	70,947	1991–2000	1.03(1.00,1.07)	Cohort	China National Hypertension follow-up survey
Japan	15	Katanoda et al. 2011	421(mortality)	63,520	1983–1995	1.24(1.12,1.37)	Cohort	Three Prefecture Cohort
Japan, Philippines, Korea, Singapore, Vietnam	16	Yorifuji et al. 2015	4,101(incidence)	50,756,699	2009-2011	1.14(1.04,1.23)	Cohort	
China	17	Guo et al.2016	368,762 (incidence)	——	1990-2009	1.07(1.06,1.09)	Cohort	National Cancer Registration of China

### Overall meta-estimates for PM2.5

**Figure 2:**
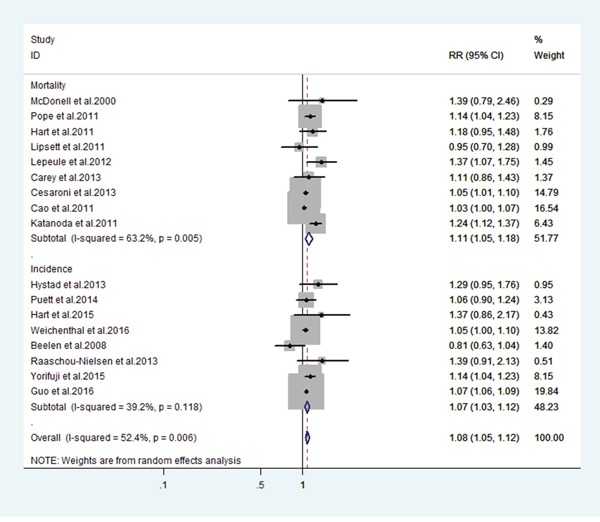
Estimates of lung cancer risk associated a 10-ug/m^3^ change in exposure to PM2.5

Figure 2 shows the estimated effect for each study, grouped by the health events associated with PM2.5. All estimates represent the change in risk of LC mortality and incidence associated with a 10 μg/m^3^ increase in PM2.5. The meta-relative mortality [95% confidence interval (CI)] for LC associated with PM2.5 was 1.11 (95% CI: 1.05, 1.18) and the meta-relative incidence was 1.08 (95% CI: 1.03, 1.12). The between-study variance for mortality and incidence was 63.2% and 39.4% of the total variance, respectively.

### Subgroup analyses

Tables 2 and 3 show the subgroup analyses by health events. Table 2 shows the association between PM2.5 and LC mortality, estimated by continent, country, gender and smoking status. Table 3 shows the association between PM2.5 and LC incidence, estimated by continent, country, gender and smoking status. In addition, Table 4 presents the subgroup analyses of full meta-estimates of combined mortality and incidence. The meta-estimate for LC risk associated with PM2.5 in mortality was 1.11 (95% CI: 1.05, 1.18), the full meta-estimate was 1.09 (95% CI: 1.05, 1.12), and in LC incidence was 1.08 (95% CI: 1.03, 1.12).

**Table 2:** Estimates for the relationship between a 10-ug/m^3^ change in PM2.5 exposure and lung cancer mortality

Exposure	RR(95%CI)	I^2^(P-Value)	Studies included (by ID)
Mortality	1.11(1.05,1.18)	63.2%(0.005)	1,2,3,4,5,11,12,14,15
Continent			
North America	1.15(1.07,1.24)	0.0%(0.406)	1,2,3,4,5
Europe	1.05(1.01,1.10)	0.0%(0.673)	11,12
Asia	1.12(0.94,1.35)	91.5%(0.001)	14,15
Country			
Developed Country	1.14(1.06,1.23)	55.2%(0.029)	1,2,3,4,5,11,12,15
Developing Country	1.03(1.00,1.07)	——	14
Gender			
Male	1.26(1.15,1.40)	0.0%(0.420)	1,15
Female	1.17(0.98,1.39)	——	15
Smoking Status			
Never	1.16(1.02,1.33)	0.0%(0.863)	5,15
Former	1.46(0.84,2.55)	75.0%(0.045)	5,15
Current	1.33(1.20,1.49)	0.0%(0.612)	5,15

**Table 3:** Estimates for the relationship between a 10-ug/m^3^ change in PM2.5 exposure and lung cancer incidence

Exposure	RR(95%CI)	I^2^(P-Value)	Studies included (by ID)
Incidence	1.07(1.03,1.12)	39.2%(0.118)	6,7,8,9,10,13,16,17
Continent			
North America	1.06(1.01,1.11)	0.0%(0.410)	6,7,8,9
Europe	1.03(0.61,1.75)	78.3%(0.032)	10,13
Asia	1.09(1.03,1.15)	53.1%(0.144)	16,17
Country			
Developed Country	1.07(0.96,1.20)	43.0%(0.118)	6,7,8,9,10,13
Developing Country	1.07(1.06,1.09)	——	17
Gender			
Male	1.23(0.83,1.81)	73.5%(0.052)	6,17
Female	1.15(1.12,1.18)	0.0%(0.917)	6,17
Smoking Status			
Never	1.10(0.76,1.59)	0.0%(0.733)	6,7
Former	1.19(0.95,1.50)	22.7%(0.255)	6,7
Current	1.03(0.87,1.21)	0.0%(0.550)	6,7

**Table 4:** Estimates for the relationship between a 10-ug/m^3^ change in PM2.5 exposure and lung cancer risk

Exposure	RR(95%CI)	I^2^(P-Value)	Studies included (by ID)
Full meta-estimate	1.08(1.05,1.12)	52.4%(0.006)	All
Continent			
North America	1.11(1.05,1.18)	26.8%(0.205)	1,2,3,4,5,6,7,8,9
Europe	1.03(0.89,1.20)	49.6%(0.114)	10,11,12,13
Asia	1.09(1.04,1.15)	80.7%(0.001)	14,15,16,17
Country			
Developed Country	1.11(1.06,1.17)	49.7%(0.015)	1,2,3,4,5,6,7,8,9,10,11,12,13,15
Developing Country	1.05(1.01,1.10)	80.3%(0.024)	14,17
Gender			
Male	1.21(1.01,1.44)	82.2%(0.001)	1,6,15,17
Female	1.15(1.12,1.18)	0.0%(0.975)	6,15,17
Smoking Status			
Never	1.16(1.02,1.30)	0.0%(0.973)	5,6,7,15
Former	1.32(1.02,1.69)	54.5%(0.086)	5,6,7,15
Current	1.20(1.01,1.41)	58.5%(0.065)	5,6,7,15

Subgroup analyses by continent showed that the mortality meta-estimates for North America, Europe and Asia were 1.15 (95% CI: 1.07, 1.24), 1.05 (95% CI: 1.01, 1.10), and 1.12 (95% CI: 0.94, 1.35), respectively. The incidence meta-estimates for North America, Europe and Asia were 1.06 (95% CI: 1.01, 1.11), 1.03 (95% CI: 0.61, 1.75), and 1.09 (95% CI: 1.03, 1.15), respectively. The full meta-estimates for North America, Europe and Asia were 1.11 (95% CI: 1.05, 1.18), 1.03 (95% CI: 0.89, 1.20), and 1.09 (95% CI: 1.04, 1.15), respectively.

Meta-estimates for the country subgroups showed that the mortality meta-estimate for developed countries was 1.14 (95% CI: 1.06, 1.23), and for developing countries was 1.03 (95% CI: 1.00, 1.07). Estimates for developing countries were only available from one study (Cao et al. 2011). The incidence meta-estimate for developed countries was 1.07 (95% CI: 0.96, 1.20), similar to developing countries which was also 1.07 (95% CI: 1.06, 1.09). Estimates for developing countries were only available from one study (Guo et al. 2016). The full meta-estimate for developed countries was 1.11 (95% CI: 1.06, 1.17), and for developing countries was 1.05 (95% CI: 1.01, 1.10).

We also conducted subgroup analyses of males and females. The meta-estimate for LC mortality associated with PM2.5 was 1.26 for males and (95% CI: 1.15, 1.40) and 1.17 for females (95% CI: 0.98, 1.39). Estimates for females were only available from one study (Katanoda et al. 2011). The meta-estimate for LC incidence associated with PM2.5 was 1.23 for males and (95% CI: 0.83, 1.81) and 1.15 for females (95% CI: 1.12, 1.18). The full meta-estimate for LC risk associated with PM2.5 was 1.21 for males (95% CI: 1.01, 1.44) and was 1.15 for females (95% CI: 1.12, 1.18).

Subgroup analyses of smoking status showed that the meta-estimate for LC mortality associated with PM2.5 in former smokers was 1.46 (95% CI: 0.84, 2.55), in current smokers was 1.33 (95% CI: 1.20, 1.49), and in never smokers was 1.16 (95% CI: 1.02, 1.33), respectively. The meta-estimate for LC incidence associated with PM2.5 in former smokers was 1.19 (95% CI: 0.95, 1.50), in never smokers was 1.10 (95% CI: 0.76, 1.59), and in current smokers was 1.03 (95% CI: 0.87, 1.21). The full meta-estimate for LC risk associated with PM2.5 in former smokers was 1.32 (95% CI: 1.02, 1.69), in current smokers was 1.20 (95% CI: 1.01, 1.41), and in never smokers was 1.16 (95% CI: 1.02, 1.30).

### Sensitivity analysis and publication bias evaluation

**Figure 3:**
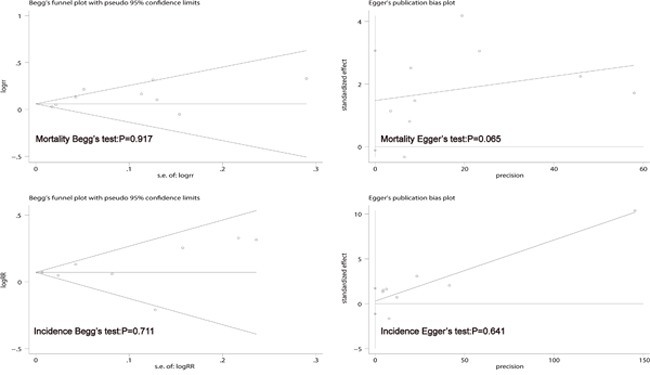
Begg's funnel plot and Egger's test to evaluate the publication bias for mortality and incidence

The sensitivity analysis showed that when the study by Katanoda et al. 2011 was omitted, the I-squared changed from 63.2% (*P* = 0.005) to 41.8% (*P* = 0.100), and the pooled RR of the remaining studies on LC mortality associated with PM2.5 was more robust and stable. The article by Katanoda et al. 2011 was a cohort study, where the heterogeneity was explained by HR. However, we used RR instead of HR in our meta-analysis. Our study showed no significant heterogeneity between LC incidence and PM2.5, thus the sensitivity analysis was omitted. Begg's funnel plot and Egger's publication bias plot were used to evaluate publication bias in relation to LC mortality/incidence and PM2.5 (Figure 3), respectively. No evidence of publication bias was found for mortality (Begg's test: *P* = 0.917; Egger's test: *P* = 0.065) or incidence (Begg's test: *P* = 0.711; Egger's test: *P* = 0.641).

## DISCUSSION

We calculated meta-estimates for the relationship between PM2.5 and LC mortality, incidence and combined incidence and mortality. Our findings suggest that long-term exposure to PM2.5 is significantly associated both with LC incidence and mortality. The association between PM2.5 and LC mortality was stronger than that between PM2.5 and LC incidence. The associations remained when analyses were restricted to continent, developed country, gender, and smoking status. In developing countries, the association between PM2.5 and LC incidence was stronger than that between PM2.5 and LC mortality. However, estimates for incidence in developing countries were only available in one study. In addition, in the full meta-estimates group, North America, developed countries, males and former smokers were suggested as risk factors for LC in those exposed to PM2.5. However, these associations lacked strong evidence as the analyses were restricted to raw data and a small number of studies.

When adjusted for continent, we found that the association between PM2.5 and LC was greater in North America than in Europe or Asia according to the mortality and full meta-estimates. The association between PM2.5 and LC was greater in Asia than in North America and Europe according to incidence meta-estimates. This study showed that although air pollution levels have declined in Western Europe and North America since the mid 20th century, they are increasing in certain other regions, especially in those regions undergoing rapid economic growth [24]. Another study found greater effect estimates in eastern than western communities, suggesting a regional pattern in the effect of exposure to PM2.5 [25]. These studies showed conflicting data concerning the association between PM2.5 and LC mortality, and the association between PM2.5 and LC incidence.

When adjusted for developed country and developing country, the meta-estimates suggested that developed countries may have an elevated mortality due to LC associated with PM2.5 compared to developing countries. According to some studies, the overall level of PM2.5 in developed countries is lower than that in developing countries, and this pattern is different from our results, perhaps due to a lack of information in developing countries.

In addition, meta-estimates suggested that males may have an elevated risk of LC associated with PM2.5 compared to females, according to the mortality, incidence and full meta-estimates. This is presumably because men may have more exposure to tobacco smoke. In our meta-analysis, one study investigated the risk of LC according to gender, and showed that males had a higher LC risk [26]. Another study demonstrated an increased risk of LC in male cooks, but not in female cooks. However, there is evidence that LC risk in cooks may be confounded by smoking [27]. However, two studies, one on males and one on females, were included in this meta-analysis and contributed to the overall meta-estimates for LC mortality. With regard to LC incidence, two studies were conducted on males (I^2^: 73.5%) and two studies on females, which contributed to the overall meta-estimates. These results are limited by a lack of sufficient studies, and we were unable to unravel the effects of PM2.5 on LC risk by gender.

In order to adjust for smoking status, some studies provided analyses of PM2.5 by smoking subgroups, allowing the assessment–differential effects of PM2.5 by smoking status (never, former, and current smokers). A comprehensive analysis of 26,957 patients with non-small cell lung cancer (NSCLC) showed that smoking status was an independent prognostic factor in NSCLC [28]. Meta-estimates from these studies suggested that former smokers may have an elevated risk of LC associated with PM2.5 compared to current smokers and never smokers in the mortality, incidence and full meta-estimates. In contrast, the never smokers showed the lowest association between PM2.5 and LC in the mortality group, and in the incidence group, the lowest association was found in current smokers. However, limited data were available to examine the relationship between PM 2.5 and LC risk as only two studies provided subgroup-specific information on smoking status in both the mortality and incidence groups. These results are limited by a lack of detailed information on patterns of former smoking. We were unable to determine the effects of PM2.5 on LC between former heavy and former light smokers, which might be expected to differ.

Certain limitations in our study may have influenced the results. Firstly, some sample sizes in the subgroups were too small. Secondly, some of the documents quoted were old. Thirdly, the mortality analysis showed significant heterogeneity, and some subgroups also reported significant heterogeneity mainly due to small sample size. Fourthly, the results of the pooled data in several eligible studies were too limited to evaluate the association between PM2.5 and LC.

## MATERIALS AND METHODS

### Search strategy

We identified potentially relevant studies by searching the PubMed, Ovid, Embase and Cochrane databases from their inception up to August 2016. Search terms included “particulate air” OR “particulate matter” OR “air pollution” And “neoplasms” or “cancer” with the results restricted to human studies. Titles and abstracts identified by electronic searches were examined by two independent reviewers, to select potentially relevant studies. In addition, the references from relevant original papers and review articles were scrutinized to obtain other pertinent publications. We considered all available cohort and case–control studies that provided information on the incidence of LC, and where the resources needed for participation were available.

### Inclusion and exclusion criteria

Eligible articles included studies which evaluated the association between PM2.5 and LC incidence and mortality. Articles were considered for inclusion in the current meta-analysis if they provided quantitative estimates of the change in LC incidence or mortality associated with exposure to PM2.5, and reported the odds ratio [OR] or hazard ratio [HR] or relative risk [RR], and its 95% confidence interval (CI) of LC associated with long-term exposure to PM2.5. Where multiple publications included overlapping study populations, the publication with the longest follow-up period was selected. Studies with poor quality and/or insufficient data, and duplicate publications were excluded from the analysis.

### Data extraction

A double abstraction process was performed for data extraction (by Feifei Huang and Liying Chen). Disagreements were resolved by consulting a third reviewer. The following data were collected from eligible studies: the first author, publication year, study region, number of LC patients exposed to PM2.5, incidence (mortality), study period, study source, gender distribution, country, smoking status, experimental method and primary outcomes (RR/HR with its 95% CI of incidence and mortality). If any of the above information was not available in the study, the authors were contacted by email for additional information. Where two or more publications included overlapping cases, we included the publication which had the largest number and/or longest follow-up.

### Quality assessment

The quality of each study was evaluated using a 9-point scoring system according to the Newcastle–Ottawa Scale (NOS) checklist for cohort and case-control studies [6] (Supplementary Table 1 and Table 2). The total score was 9, and a high-quality study in our analysis was defined as a study with at least 7 points.

### Statistical analyses

A dose-response meta-analysis was performed to examine the relationship between PM2.5 and LC. All study estimates were converted to represent the change in LC incidence or mortality per 10-μg/m^3^ unit increase in exposure to PM2.5. In this meta-analysis, the relative risks (RRs) and 95%CIs were considered the effect size for all studies. Because the incidence of LC was low, the HR from cohort studies was approximated to RR. We reported all risk estimates produced by the current meta-analysis as RR for simplicity. As significant heterogeneity would contribute to the variance, the pooled RRs for specific PM2.5 values were estimated using a random-effects model. Subgroup analyses were performed according to continent, country, gender, and smoking status, respectively. Furthermore, sensitivity analysis was performed to assess the source of heterogeneity. Potential publication bias was detected using Begg's test and Egger's test. Two-tailed *P* values < 0.05 were considered statistically significant. All statistical analyses were performed using Stata 12.0 software.

## CONCLUSIONS

In conclusion, the relative risks of LC incidence and mortality following exposure to PM2.5 were 1.08 (95% CI: 1.03, 1.12) and 1.11 (95% CI: 1.05, 1.18), respectively. The findings from the present meta-analysis may provide some evidence for policy makers and public health practitioners worldwide.

## SUPPLEMENTARY TABLES


