# Supplementary material for: Relationship between exposure to PM2.5 and lung cancer incidence and mortality: A meta-analysis
Source: Oncotarget. 2017 Apr 21;8(26):43322–31. doi: 10.18632/oncotarget.17313 (PMC5522148; doi:10.18632/oncotarget.17313)
Supplement: Supplementary file 1 [file oncotarget-08-43322-s001.pdf]

Relationship between exposure to PM2.5 and lung cancer incidence and mortality: A meta-analysis

SUPPLEMENTARY TABLES

Supplementary Table 1: Newcastle-ottawa quality assessment scale for cohort studies in the meta-analysis

NEWCASTLE - OTTAWA QUALITY ASSESSMENT SCALE COHORT STUDIES									
	Selection				Comparability	Outcome			
Study	Representativeness of the exposed cohort	Selection Of the Non-exposed cohort	Ascertainment of exposure	Demonstration that outcome of interest was not present at start of study	Comparability of cohorts on the basis of the design or analysis	Assessment of outcome	Was follow-up long enough for outcomes to occur	Adequacy of follow up of cohorts	Total score
McDonnell et al.2000	1	1	1	0	2	1	1	1	8
Pope et al.2011	1	1	1	0	1	1	0	0	5
Hart et al. 2011	0	1	1	0	2	1	0	0	5
Lipsett et al. 2011	0	1	1	0	2	1	0	0	5
Lepeule et al. 2012	1	1	1	0	1	1	1	1	7
Puett et al. 2014	0	1	1	0	2	1	1	1	7
Hart et al.2015	1	1	1	1	2	1	0	0	7
Weichenthal et al.2016	1	1	1	0	1	1	0	0	5
Beelen et al. 2008	1	1	1	0	2	1	0	0	6
Carey et al. 2013	1	1	1	0	2	1	0	0	6
Cesaroni et al. 2013	1	1	1	0	2	1	0	0	6
Raaschou-Neilsen et al. 2013	1	1	1	0	2	1	0	0	6
Cao et al. 2011	1	1	1	0	2	1	1	1	8
Katanoda et al. 2011	1	1	1	0	2	1	1	1	8
Yorifuji et al. 2015	1	1	1	0	1	1	0	0	5
Guo et al.2016	1	1	1	0	1	1	0	0	5

Supplementary Table 2: Newcastle-ottawa quality assessment scale for case-control study in the meta-analysis

NEWCASTLE - OTTAWA QUALITY ASSESSMENT SCALE CASE CONTROL STUDIES									
			Selection		Comparability		Exposure		
Study	Is the case definition adequate	Representativeness Of the cases	Selection of Controls	Definition of Controls	Comparability of cases and controls on the basis of the design or analysis	Ascertainment of exposure	Same method of ascertainment for cases and controls	Non-Response rate	Total score
Hystad et al. 2013	1	1	1	1	2	1	1	0	8
